# Stress management training program to address caregiver burden and perceived stress among family caregivers of patients undergoing hemodialysis: a randomized controlled trial study

**DOI:** 10.1186/s12882-024-03795-5

**Published:** 2024-10-14

**Authors:** Ramisa Khouban-Shargh, Seyedmohammad Mirhosseini, Saeed Ghasempour, Mohammad Hasan Basirinezhad, Ali Abbasi

**Affiliations:** 1 Center for Health Related Social and Behavioral Sciences Research, Shahroud University of Medical Sciences, Shahroud, Iran; 2 Department of Nursing, School of Nursing and Midwifery, Shahroud University of Medical Sciences, Shahroud, Iran; 3 Student Research Committee, School of Nursing and Midwifery, Shahroud University of Medical Sciences, Shahroud, Iran; 4 Department of Epidemiology and Biostatistics, School of Public Health, Shahid Sadoughi University of Medical Sciences, Yazd, Iran

**Keywords:** Caregiver burden, Perceived stress, Stress management, Hemodialysis

## Abstract

**Background:**

The objective of the current study was to assess the effectiveness of stress management training, grounded in Lazarus and Folkman’s stress management model, on reducing caregiving burden and perceived stress among family caregivers of patients on hemodialysis.

**Methods:**

This two-group clinical trial study was conducted in parallel design among 60 family caregivers of patients on hemodialysis in 2023. The participants were divided into two groups of training and control using a random quadruple block allocation method. The intervention took place over two months, in six online group sessions of 35–45 min. Zarit Burden Inventory (ZBI) and Cohen’s Perceived Stress Scale (PSS-14) were used to collect information before and two weeks after the intervention. The study data were analyzed using and analysis of covariance (ANCOVA), pair, and independent t-tests at a significance level of 0.05.

**Results:**

At baseline, the two groups exhibited homogeneity in terms of mean scores for caregiving burden (Training group = 50.8 ± 4.9; Control group = 49.1 ± 6.0; *P =* 0.264) and perceived stress (Training group = 32.8 ± 4.7; Control group = 31.5 ± 2.4; *P =* 0.192). Nevertheless, following the intervention, there was a significant decrease in caregiving burden (Training group = 45.9 ± 4.1; Control group = 49.0 ± 5.8; *P =* 0.017) and perceived stress (Training group = 28.0 ± 4.4; Control group = 30.7 ± 3.5; *P =* 0.01) scores within the training group compared to the control group.

**Conclusion:**

Based on the findings of the current study, given that family caregivers of patients on hemodialysis encounter psychological distress and contend with the negative aspects of care, it is advisable to implement psycho-educational interventions, such as stress management training. Incorporating these interventions into the care plan for hemodialysis could help mitigate these adverse consequences and provide valuable support for family caregivers.

**Trial registration:**

Iranian Registry of Clinical Trials (IRCT), IRCT20180728040617N6. Registered on 17/04/2023.

**Supplementary Information:**

The online version contains supplementary material available at 10.1186/s12882-024-03795-5.

## Background

Chronic kidney disease (CKD) is a prevalent global health issue, signifying an irreversible deterioration in kidney function [1, 2]. Treatment options for CKD include hemodialysis (HD), peritoneal dialysis (PD), and kidney transplantation (KT) [3]. Hemodialysis is the most widely utilized treatment method globally, as well as in Iran [4–6]. While essential for patient survival and longevity, studies have revealed that hemodialysis can be associated with side effects such as fatigue, low blood pressure, cramps, confusion, and lifestyle adjustments for patients [2]. Furthermore, individuals undergoing hemodialysis experience a range of physical, psychological, and social stressors [7], leading to significant disability, loss of function, and increased dependency on family caregivers [8].

Family caregivers are critical figures in providing daily psychological and emotional support to the patient, serving as the linchpin of the care system [9]. They engage significantly in patient care and aid individuals in managing and adapting to chronic diseases [10]. Despite their pivotal role, these caregivers may face physical and psychological vulnerability, and if the patient’s needs are not addressed, they can experience exhaustion, leading to a decline in their caregiving ability [11]. Studies conducted by Jafari et al. (2018) revealed that 80.1% of family caregivers of patients on hemodialysis experience moderate to severe levels of caregiver burden [12], whereas Menati et al. (2020) found that 86.0% of these caregivers report high levels of caregiver burden [13].

The term “caregiver burden” encompasses the physical, emotional, and economic challenges encountered by caregivers and can be categorized into objective and subjective components. Objective caregiver burden pertains to the shifts and disruptions in various aspects of the caregiver’s life. In contrast, subjective caregiver burden refers to the caregiver’s emotional and attitudinal responses to the care experience [14]. This concept tends to intensify over time as the patient’s condition deteriorates. Consequently, caregivers may face health issues, social isolation, family relationship strains, inadequate patient care, disruptions in daily routines and recreational activities, sleep disturbances, decreased quality of life, and caregiver burnout. Ultimately, this weight may lead to caregiver abandonment of the patient [15].

Numerous studies have indicated that a noteworthy portion of caregivers of patients on hemodialysis contend with various psychological challenges [16–18]. According to Tao et al. (2023), the mental well-being of these patients and their caregivers is correlated [18]. Consequently, the psychological issues faced by patients undergoing hemodialysis are closely linked with the psychological challenges experienced by their caregivers [16]. One particular psychological issue is perceived stress, which encompasses feelings of confusion or uncertainty when evaluating the possibility of a stressful event [19]. The physical and mental state of patients undergoing hemodialysis, along with their prolonged treatment, contributes to the stress experienced by their family caregivers [20]. Tao et al. (2023) found that 68.3% of patients undergoing hemodialysis and 66.8% of their caregivers experienced high levels of perceived stress [18]. These findings underscore the pressing need for implementing varied solutions to mitigate the perceived stress and caregiver burden experienced by these patients’ family caregiver.

Stress management training has become increasingly prominent and offers a potential solution to address these challenges. This kind of program equips individuals with the tools to effectively deal with stress and mitigate its adverse impacts by understanding its biological origins. By acquiring life skills, individuals can shield themselves from stress, while also developing management strategies to minimize its negative aspects [21]. While eliminating stress may be unattainable, people can learn to effectively manage it. Stress management involves the ability, capacity, and skill to recognize, assess, and regulate one’s and others’ emotions. In this framework, it is critical to employ preventative approaches, focusing on early detection of stress and implementing effective coping mechanisms. Stress management training, as a life skill, enhances individuals’ adaptive capacity, as well as their psychological and social abilities to navigate life’s challenges and reduce stress. Furthermore, it enables individuals to translate knowledge, values, and attitudes into practical skills, exerting a meaningful influence on their self-perception and relationships with others. This comprehensive program encompasses various elements, including raising awareness about stress, problem-solving training, instruction in self-expression skills, anger management, self-regulation, and activity planning [22].

It’s crucial to recognize that family caregivers of patients on hemodialysis are exceptionally vulnerable both physically and mentally due to the demanding nature of patient care, which can lead to physical, mental, and emotional exhaustion [23]. These caregivers contend with high levels of caregiver burden and perceived stress. Consequently, it’s essential to introduce effective strategies to alleviate the caregiver burden and perceived stress experienced by these caregivers. Stress management training emerges as a potential solution, offering the prospect of mitigating the caregiver burden and perceived stress. Hence, this study was designed and conducted to assess the efficacy of stress management training programs on the caregiver burden and perceived stress among family caregivers of patients undergoing hemodialysis.

## Methods

### Study design and settings

This parallel randomized clinical trial (with clinical trial code IRCT20180728040617N6) was conducted on family caregivers of patients on hemodialysis referred to Imam Hossein Hospital and Red Crescent Hemodialysis Center in Shahroud, Iran. The study took place between August and November 2023. Sampling was performed using a convenience technique. Inclusion criteria required a minimum level of literacy, at least six months of experience in patient care [24, 25], being a member of the patient’s family (one family caregiver for each patient, selected based on self-reported most frequent daily interaction with the patient), and access to a smartphone for communication during stress management training. Participants were excluded from the study if they were employed as healthcare staff, or participated in other supportive interventions. Following previous research [26], participants in the training group were considered dropouts if they missed more than two sessions. All participants complied with the maximum allowed absence, so all were included in the post-test evaluation.

**Fig. 1:**
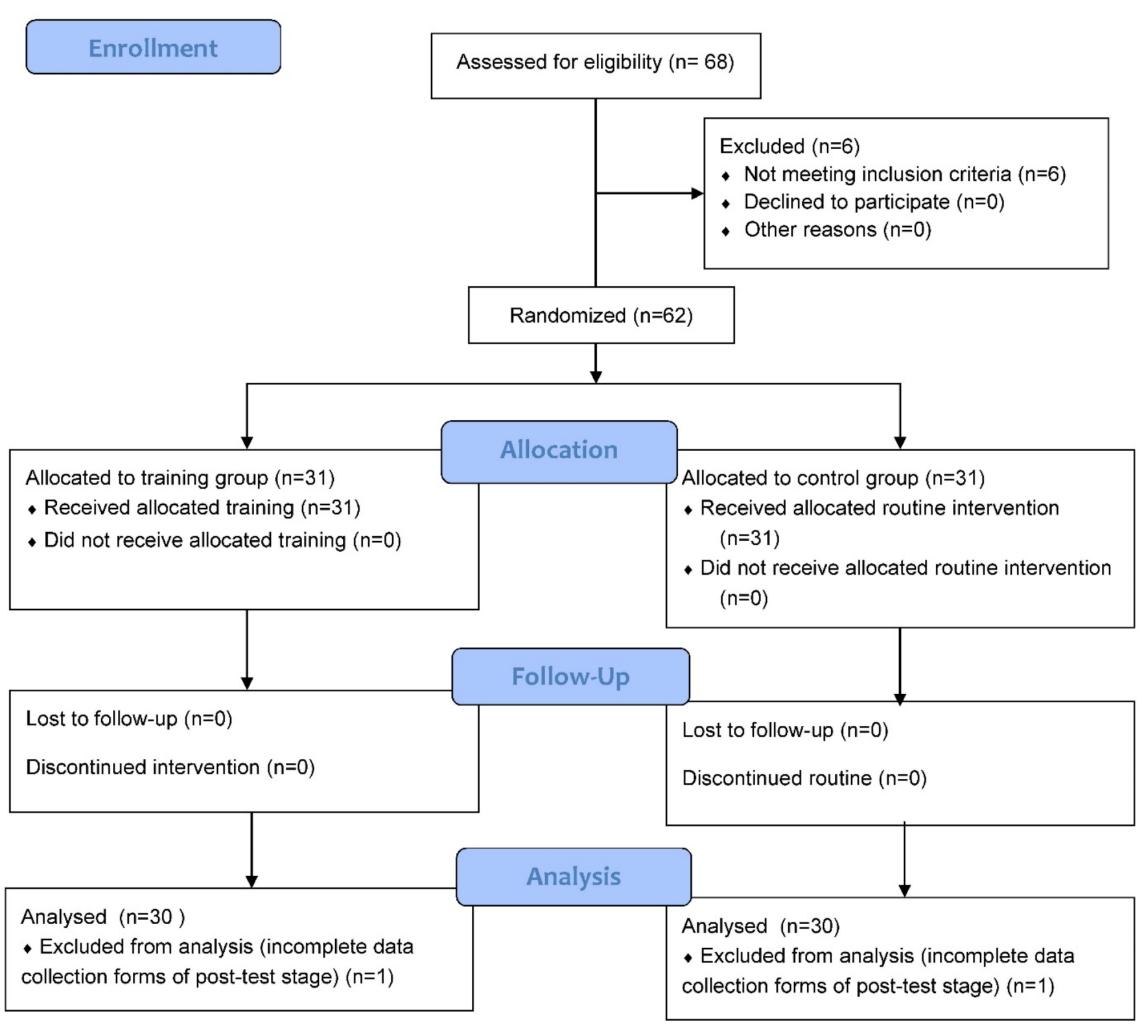
CONSORT flow diagram of the study

The medical records of all referred patients included contact numbers for their family caregivers. Initial arrangements and conditions for caregiver participation in the study were assessed by phone contact. Additionally, patients were accompanied by their family caregivers during their visits to the medical centers for hemodialysis. Through these two methods, the researcher was able to communicate with the patients’ family caregivers. Two participants, one from the control group and one from the training group, had incomplete post-test data collection forms. These participants were excluded from the data analysis. The allocation procedure for this study was based on the random allocation of quadruple blocks generated using SPSS software. (Fig. 1)

### Intervention

After securing the necessary permits, the study’s objectives were communicated to all participants, and their verbal and written informed consent was obtained for study participation. The intervention sessions were conducted on the WhatsApp social network, based on the stress management model of Lazarus and Folkman (1984). The primary goal of this model was to enhance family caregivers’ ability to manage stressful situations during patient care. The model generally encompassed four stages: (1) Defining stress and its implications, (2) Identifying inefficient and stress-inducing cognitions, (3) Replacing inefficient thoughts with realistic cognitions and re-evaluating them, and (4) Employing appropriate coping strategies (emotion and problem-oriented based copings) along with problem-solving skills [27].

The intervention was conducted by the first author, a trained clinical psychologist, over six weeks comprising six online group sessions (weekly sessions). These sessions incorporated group discussions, question-and-answer segments, and the sharing of caregiving experiences. To ensure focused communication, participants were briefed on the rules of online group participation, emphasizing the sharing of messages solely related to caregiving topics and the needs of their patients, while refraining from unrelated discussions. Each session lasted between 35 and 45 min. Moreover, a survey was carried out among the participants in each group to schedule daily support sessions in the form of online group video calls, ensuring their attendance at pre-arranged times. The stress management training program in this study was personalized to address the specific background of hemodialysis, its consequences, and the caregivers’ needs. The psychological strategies underpinning the intervention were centered on correcting cognition and implementing effective coping skills and problem-solving techniques. Additionally, family caregivers were encouraged to share their successful patient care experiences in the online group chats. Family caregivers engaged in a supportive environment, where they could openly ask and answer questions and discuss their caregiving challenges and issues in a friendly setting. To enhance learning, visual aids such as pictures, slides, and video tutorials were provided to demonstrate care techniques. The research team maintained continuous communication with the family caregivers, allowing them to share their concerns at any time of day. Furthermore, a direct telephone connection was established between family caregivers with one of the researchers to reach out in case of specific problems or questions. The information presented above adheres to the TIDieR checklist, a tool employed for organizing details pertaining to behavioral interventions [28].

In contrast, participants in the control group received routine interventions in medical centers. Upon completion of the study, the intervention program was subsequently carried out for the control group.

### Data collection

The data collection tools consisted of a demographic profile form, encompassing questions related to age, gender, education level, marital status, employment status, relationship with the patient, and the duration of patient care. Additionally, the Zarit Burden Inventory (ZBI) and Cohen’s Perceived Stress Scale (PSS-14) were administered before and two weeks after the intervention.

The Zarit Burden Inventory (ZBI), designed by Zarit in 1980, was implemented in the form of an interview with family caregivers of the patients in this study to assess the level of caregiver burden. The questionnaire comprises 22 questions focusing on the burden experienced by caregivers due to patient care. Responses are based on a Likert scale ranging from ‘never’ (0) to ‘always’ (4). Scores below 30 suggest a mild burden, while scores between 31 and 60 indicate a moderate burden, and scores between 61 and 88 indicate a severe caregiver burden. Each participant’s score can range from 0 to 88, with a higher score showing a greater burden of care [29]. It’s worth noting that the internal consistency of the Persian version of the ZBI was assessed at an acceptable level with a Cronbach’s alpha coefficient of 0.85 in the study conducted by Mousaei et al. (2023) [24], while in the current study, the Cronbach’s alpha coefficient for this inventory was found to be 0.72.

The 14-item perceived stress scale was developed by Cohen et al. (1983). For each item, scores range from zero to four. Therefore, the highest and lowest scores for this scale can be 56 and zero, respectively. Notably, questions 4, 5, 6, 7, 9, 10, and 13 are scored inversely [19]. In a previous study, the scale demonstrated favorable internal consistency, with a Cronbach’s alpha coefficient of 0.73 [26]. The Cronbach’s alpha coefficient for this study was also reported as 0.76.

### Sample size

The present study’s sample size was determined based on Ata et al.‘s research (2018) [30], considering the mean and standard deviation reported for the caregiver burden variable. A confidence level of 95% and a power of 80% were taken into account. Anticipating potential sample attrition, the sample size was estimated at 30 individuals (totaling 60 family caregivers across two groups). Given the nature of the intervention, participant blinding was not feasible. However, the data collector and statistical consultant were blinded.

### Statistical analysis

In the analysis, family caregivers were considered as the smallest unit. Descriptive data, including frequency and percentage, were utilized to present demographic characteristics (gender, marital status, education level, occupational status, and patient relationship). Mean and standard deviation were used to describe age, duration of patient care, caregiver burden, and perceived stress. To delineate differences between the two groups, the chi-square test and Fisher’s exact test were employed. Furthermore, an independent t-test was utilized to illustrate the difference between the two groups regarding the mean scores of caregiver burden and perceived stress. The paired t-test was utilized to illustrate the statistical difference in intra-group changes. Lastly, the analysis of covariance (ANCOVA) model was applied to compare post-intervention caregiver burden and perceived stress scores between the control and training groups, while factoring in and eliminating the influence of the pre-test score and group variable. The significance level for all analyses was set at 0.05, and all statistical analyses were carried out using the Statistical Package for Social Sciences (SPSS) and STATA software.

### Ethical considerations

The family caregivers’ participation in the current study was voluntary, and they were provided with clear information about the study’s objectives and the confidentiality of their information from the beginning. Data analysis and publication were conducted anonymously, in strict adherence to ethical considerations. Both written and verbal informed consent were obtained from all participants. All procedures were conducted in accordance with the autonomy of both patients and their family caregivers.

This study received approval from the Biomedical Research Ethics Council of Shahroud University of Medical Sciences under the ethical code IR.SHMU.REC.1401.218. Furthermore, the authors followed the principles of the Committee on Ethics in Publication (COPE) when publishing the findings. It’s also worth noting that the content of the intervention was subsequently implemented in the control group after the completion of the study.

## Results

The results from the present study revealed that the average age in the training group at 37.8 ± 11.8, while in the control group, it was 41.0 ± 11.1. Notably, the two groups exhibited homogeneity in their demographic distribution, encompassing factors such as gender, marital status, education, employment, monthly income, relationship with the patient, presence of underlying disease, and the duration of care given to the patient, with no statistically significant differences between them (*P >* 0.05). Additional findings are shown in Table 1.

**Table 1:** Demographic characteristics of caregivers

Variables		Groups		*P*-value
		Training	Control	
		*n* (%)	*n* (%)	
Gender	Male	11 (36.7)	16 (53.3)	0.299*
	Female	19 (63.3)	14 (46.7)	
Marital status	Married	18 (60.0)	21 (70.0)	0.600*
	Single	10 (33.3)	7 (23.3)	
	Divorced	1 (3.3)	2 (6.7)	
	Deceased wife	1 (3.3)	0 (0.0)	
Level of education	Elementary school	1 (3.3)	0 (0.0)	0.157*
	Cycle	4 (13.3)	6 (20.0)	
	Diploma	9 (30.0)	7 (23.3)	
	Associate degree	5 (16.7)	10 (33.3)	
	BSc	11 (36.7)	5 (16.7)	
	MSc	0 (0.0)	2 (6.7)	
Employment status	Unemployed	2 (6.7)	1 (3.3)	0.335*
	Housewife	12 (40.0)	9 (30.0)	
	Self-employed	4 (13.3)	8 (26.7)	
	Employee	9 (30.0)	8 (26.7)	
	Retired	1 (3.3)	4 (13.3)	
	Student	2 (6.7)	0 (0.0)	
Relationship with the patient	Father	1 (3.3)	0 (0.0)	0.940*
	Mother	2 (6.7)	2 (6.7)	
	Child	13 (43.3)	10 (33.3)	
	Wife/husband	12 (40.0)	16 (53.3)	
	Sister	1 (3.3)	1 (3.3)	
	Other	1 (3.3)	1 (3.3)	
Underlying disease	No	23 (76.7)	20 (66.7)	0.567*
	Yes	7 (23.3)	10 (33.3)	
Income	Low	18 (60.0)	20 (66.7)	0.855*
	Medium	9 (30.0)	8 (26.7)	
	High	3 (10.0)	2 (6.7)	
		Mean ± SD	Mean ± SD	
Age (year)		37.8 ± 11.8	41.0 ± 11.1	0.674**
Duration of caregiving (Month)		32.4 ± 36.0	25.2 ± 22.6	0.148**

* Chi-squared test

** Independent t test

n: Frequency; %: Percent; SD: Standard deviation.

Based on the results presented in Table 2, before the intervention, both groups did not exhibit a significant difference in terms of the average caregiver burden score (*P =* 0.264). However, after the intervention, the training group reported significantly lower caregiver burden scores compared to the control group (*P =* 0.017). Additionally, a decrease in the average caregiver burden score compared to before the intervention was observed in both groups; yet, notably, the reduction seen in the training group was significantly higher than that in the control group (*P <* 0.001).

**Table 2:** Mean scores of caregiver burden in caregivers of hemodialysis patients before and after intervention in both groups

Variables	Groups		Intergroup test results
	Training (*n* = 30)	Control (*n* = 30)	
	Mean ± SD	Mean ± SD	
**Pre-intervention**	50.8 ± 4.9	49.1 ± 6.0	*P* = 0.264* t = 1.1 df = 58
**Post-intervention**	45.9 ± 4.1	49.0 ± 5.8	*P* = 0.017* t=-2.4 df = 58
**Mean Differences**	-4.9 ± 3.0	-0.1 ± 1.6	*P* < 0.001* t=-7.7 df = 58
**Intragroup test results**	*P* < 0.001** t = 9.0 df = 29	*P* = 0.654** t = 0.5 df = 29	

* Independent t test

** Paired t-test

n: Frequency; P: P-value; SD: Standard deviation.

Regarding the average scores reported for perceived stress, the two groups did not exhibit a statistically significant difference before the intervention (*P =* 0.192). However, following the implementation of the stress management program, the perceived stress scores in the training group were found to be significantly lower than those in the control group (*P =* 0.01). When considering the changes in the average perceived stress score, it was evident that the training group experienced a greater decrease compared to the control group (*P <* 0.001). Additional results are presented in Table 3. Further analysis using STATA software confirmed that a power of one, indicating that the study had a high likelihood of detecting a statistically significant effect of intervention on caregiver burden and perceived stress.

**Table 3:** Mean scores of perceived stress in caregivers of patients on hemodialysis before and after intervention in both groups

Variables	Groups		Intergroup test results
	Training (*n* = 30)	Control (*n* = 30)	
	Mean ± SD	Mean ± SD	
**Pre-intervention**	32.8 ± 4.7	31.5 ± 2.4	*P* = 0.192* t = 1.3 df = 58
**Post-intervention**	28.0 ± 4.4	30.7 ± 3.5	*P* = 0.010* t=-2.7 df = 58
**Mean Differences**	-4.8 ± 3.4	-0.8 ± 1.8	*P* < 0.001* t=-5.7 df = 58
**Intragroup test results**	*P* < 0.001** t = 7.6 df = 29	*P* = 0.025** t = 2.4 df = 29	

* Independent t test

** Paired t-test

n: Frequency; P: P-value; SD: Standard deviation.

Based on the results presented in Table 4, an analysis of covariance (ANCOVA) was conducted to evaluate the factors influencing caregiver burden scores and perceived stress after the intervention. The findings revealed that the average caregiver burden score before the intervention and the group variable significantly influenced the average caregiver burden score after the intervention. Specifically, participants in the training group reported a caregiver burden score 4.478 units lower than the control group. Similarly, the perceived stress score before the intervention and the group variable showed a significant impact on perceived stress after the intervention. Notably, in the training group, the perceived stress was significantly lower by 3.710 points compared to the control group.

**Table 4:** Effect of stress management intervention on caregiver burden and perceived stress after eliminating the effect of pre-test mean scores

Variables			β	SE	t	*P*-value
**Caregiver burden**	Constant value		8.737	2.633	3.318	0.002
	Mean score of before intervention		0.820	0.053	15.489	< 0.001
	Group	Control	ref			
		Training	-4.478	0.578	-7.751	< 0.001
**Perceived stress**	Constant value		5.614	2.956	1.900	0.063
	Mean score of before intervention		0.797	0.093	8.613	< 0.001
	Group	Control	ref			
		Training	-3.710	0.687	-5.397	< 0.001

SE: Standard error.

## Discussion

The present study highlights the effectiveness of stress management training program based on the transactional model of stress coping proposed by Lazarus and Folkman (1984) in alleviating the caregiving burden and perceived stress experienced by family caregivers of patients on hemodialysis. It is important to recognize that the care of patients on hemodialysis imposes additional responsibilities on family caregivers for various reasons, including role conflicts, emotional strain, and financial burdens, thereby subjecting them to significant burdens. According to our findings, participants in both the intervention and control groups experienced moderate levels of caregiving burden (50.8 ± 4.9 and 49.1 ± 6.0, respectively). Consistent with our findings, previous studies have also demonstrated that family caregivers of patients on hemodialysis commonly experience moderate levels of caregiving burden [31–33].

Numerous studies have focused on reducing the caregiving burden for individuals caring for patients on hemodialysis, often through comprehensive interventions. It is worth noting that an effective approach to alleviating this burden involves the utilization of interventions centered around the adoption of efficient coping strategies, supportive educational programs, family-oriented programs, and psychological interventions [34, 35]. In line with this perspective, the current study employed a training program based on the transactional model of stress coping proposed by Lazarus and Folkman (1984). This program aimed to impart appropriate coping skills for effectively managing the challenges associated with patient care.

Several studies have demonstrated the efficacy of support interventions of this nature [26, 36]. According to the results of the current study, the utilization of support grounded in this model has notably reduced the caregiving burden. In this regard, Hemmati Maslakpak et al. (2019) conducted a study aiming to determine the impact of psycho-educational interventions on the caregiver burden of family caregivers of patients on hemodialysis. The results of the study showed that implementing this intervention, in the form of six group discussion sessions and four workshop sessions, plays a key role in reducing the caregiver burden of these caregivers [31]. Sotoudeh et al. (2019) also found that family-oriented educational interventions are effective in reducing the caregiver burden of family caregivers of patients on hemodialysis [37]. Similarly, Bártolo et al. (2022) stated in a systematic review that psycho-educational interventions designed to improve family caregivers’ ability to care and cope with their caregiving role are effective in reducing the burden of caregiving and improving the quality of life of these caregivers [38]. However, more studies are needed to obtain stronger evidence and confirm the aforementioned findings, especially concerning family caregivers of patients on hemodialysis. Furthermore, studies conducted in this field have confirmed the positive impact of such interventions in other communities, including family caregivers of patients with COVID-19, dementia, and breast cancer. For example, the results of Mirhosseini et al.‘s (2021) study showed that an Internet-based stress management program, based on the Lazarus and Folkman (1984) model, effectively reduced the caregiving burden among family caregivers of COVID-19 patients during the post-discharge period [39]. Moreover, Pihet et al.‘s research in 2018 indicated a significant reduction in psychological distress and caregiving burden, along with an enhancement in the self-efficacy of dementia patient caregivers following the implementation of a group psychoeducational intervention based on this model [40]. Furthermore, Gabriel et al.‘s study demonstrated a significant improvement in the quality of life and reduction of caregiving burden among caregivers of breast cancer patients following utilizing this kind of support [41]. Although the studied populations in these references may differ from the context of hemodialysis, the results align with the use of the intervention explored in the present study.

In this study, alongside psychological support, specific educational support tailored to hemodialysis was also offered. In line with this, the research by Hayati et al. (2023) demonstrated that providing training on health-promoting behaviors reduced the caregiving burden in family caregivers of patients on hemodialysis [42]. Similarly, Alnazly (2018) showed that educating caregivers about the necessary care for patients on hemodialysis led to a reduction in their burden and improved care outcomes [43]. Furthermore, the study by Bahrami et al. (2019) indicated that an educational intervention, comprising six sessions for children undergoing hemodialysis and five sessions of 45–50 min for their mothers, effectively reduced the caregiving burden among mothers and significantly decreased anxiety in children [33]. In addition, the findings of the study by Zarmohammadi et al. (2024) showed that providing self-management training according to the 5-A self-management model has led to a significant reduction in the burden of care among caregivers of patients on hemodialysis [44].

The implementation of psychoeducational interventions, in accordance with a stress management program for family caregivers, has enabled them to enhance their understanding of hemodialysis, develop problem-solving skills, and bolster their social support. This form of support has notably contributed to the improvement of family caregivers’ ability to provide care, cope with their caregiving role, reduce the burden of caregiving, and enhance their overall quality of life [38]. Utilizing technology to deliver these interventions has shown a significant positive effect on alleviating the caregiving burden. Concurrently, the implementation of group-based psychoeducational interventions has been associated with the reduction of anxiety, depression, insomnia, and an enhancement in the quality of life and self-efficacy of caregivers [45]. In line with these insights, the present study endeavored to introduce online stress management support for family caregivers in a group setting.

According to the findings of the present study, the utilization of group support training has led to a significant reduction in perceived stress scores among family caregivers. To the best of our knowledge, no study has previously assessed the impact of this form of support on the perceived stress of family caregivers within the context of hemodialysis. Most studies have been conducted in the context of other diseases. For instance, a prior study in the field of COVID-19 revealed that the implementation of a six-session stress management support program resulted in a significant reduction in perceived stress among family caregivers of COVID-19 patients [26]. Moreover, López-Liria et al. (2019) showed that the utilization of the stress management program, based on the aforementioned model, significantly reduced distress levels among parents of disabled children and improved their coping strategies in dealing with challenging situations [46]. Similarly, a Similar study by Sari et al. (2022) revealed the same results among caregivers of chronic neurological and mental patients [47]. Furthermore, online interventions providing psychosocial education to patients and their families have yielded positive results in addition to reducing caregivers’ distress and enhancing their resilience [48]. Implementing such support in an online group setting represents an economical measure for family caregivers and does not necessitate advanced facilities. In addition to the mentioned benefits, this form of support facilitates the sharing of family caregivers’ care experiences, thereby enhancing their ability to care for patients. This finding has been corroborated by studies conducted by Vaughan et al. (2018) and Tuckey et al. (2022) [49, 50].

Prior research on the studied disease, stress management program implementation models, and socio-cultural backgrounds has demonstrated variability. However, their collective findings consistently support the favorable effectiveness of this form of intervention in mitigating the negative consequences of care. As the present study was situated within the Iranian community, which harbors specific religious and social beliefs, caution should be exercised when generalizing the findings of this study to other cultures and societies. Furthermore, the data collection for this study relied on a questionnaire and a scale, so the external validity of the results is threatened by response bias. Additionally, the data collection tools were not specifically designed for the context of hemodialysis, raising the possibility of measurement error. In this study, the intervention’s effectiveness was not assessed at multiple time points, prompting the recommendation to evaluate its impact at various stages in future studies.

Acknowledging these limitations, it’s crucial to highlight the novelty of this study in incorporating the use of Lazarus and Folkman’s stress management model within the hemodialysis field and evaluating its effects on care aspects, notably perceived stress and caregiving burden. The findings bear significant implications for the clinical implementation of this type of stress management program.

## Conclusions

Family caregivers of patients on hemodialysis often experience significant mental distress and a substantial burden of care. The implementation of group support based on the stress management program is recommended as an effective intervention with an easy implementation method to alleviate the psychological and caregiving burden among family caregivers of patients on hemodialysis.

## Electronic supplementary material

Below is the link to the electronic supplementary material.

Supplementary Material 1

## Data Availability

The datasets used and/or analyzed during the current study are available from the corresponding author on reasonable request.

## Abbreviations

ZBI: Zarit Burden Inventory
CKD: Chronic Kidney Disease
PD: Peritoneal Dialysis
KT: Kidney Transplantation
PSS-14: 14 Items Perceived Stress Scale
ANCOVA: Analysis of Covariance
SPSS: Statistical Package for Social Sciences
COPE: Committee on Ethics in Publication
COVID-19: Coronavirus Disease 2019
